# Molecular and Clinical Issues about the Risk of Venous Thromboembolism in Older Patients: A Focus on Parkinson’s Disease and Parkinsonism

**DOI:** 10.3390/ijms19051299

**Published:** 2018-04-26

**Authors:** Claudio Tana, Fulvio Lauretani, Andrea Ticinesi, Beatrice Prati, Antonio Nouvenne, Tiziana Meschi

**Affiliations:** 1 Internal Medicine and Critical Subacute Care Unit, Medicine and Geriatric-Rehabilitation Department, University-Hospital of Parma, 43126 Parma, Italy; ctana@ao.pr.it (C.T.); andrea.ticinesi@unipr.it (A.T.); bprati@ao.pr.it (B.P.); ANouvenne@ao.pr.it (A.N.); tiziana.meschi@unipr.it (T.M.); 2 Department of Medicine and Surgery, University-Hospital of Parma, 43126 Parma, Italy; 3 Cognitive and Motor Center, Medicine and Geriatric-Rehabilitation Department of Parma, University-Hospital of Parma, 43126 Parma, Italy

**Keywords:** venous, thromboembolism, risk, old, frail, patients

## Abstract

Venous thromboembolism (VTE) is a common and potentially life-threatening condition which includes both deep-vein thrombosis (DVT) and pulmonary embolism (PE). VTE has a significant clinical and epidemiological impact in the elderly, and its incidence increases to more than 1% per year in older patients, suggesting the presence of specific age-related risk factors in this population. Immobilization seems to predominate as the main cause in patients admitted for medical acute illness in medicine wards, and there is evidence of a high risk in older patients with immobilization resulting from advanced forms of Parkinson’s disease (PD), regardless of the presence of an acute medical condition. In this review, we would to discuss the recent evidence on clinical, molecular and epidemiological features of VTE in older frail subjects focusing on patients with PD and parkinsonism. We also discuss some therapeutic issues about the risk prevention and we suggest a thorough comprehensive geriatric assessment that can represent an optimal strategy to identify and prevent the VTE risk in these patients.

## 1. Introduction

Venous thromboembolism (VTE) is a common and potentially life-threatening condition which encompasses both deep-vein thrombosis (DVT) and pulmonary embolism (PE). Deep veins are a common site of thrombosis, and specified risk factors can be found in over 80 percent of VTE patients [1]. Several etiologies can be associated with VTE, both inherited (e.g., factor V Leiden and protein C, S or antithrombin (AT) deficiency) and acquired such as trauma, malignancy, surgery (in particular orthopedic), immobilization and chronic diseases (e.g., heart failure, inflammatory bowel disease, myeloproliferative disorders and nephrotic syndrome). Antiphospholipid syndrome (APS) is an important additional cause of acquired thrombosis, both arterial and venous, and should be investigated particularly in female patients with unexplained thrombosis. Among acquired causes, immobilization seems to predominate in patients admitted for medical acute illness in medicine wards, but there is also an increasing evidence of a high risk in older patients with immobilization resulting from advanced forms of Parkinson’s (PD), regardless of the presence of an acute medical condition. In these patients, the presence of a systemic, chronic inflammation seems to contribute significantly to the thrombotic risk.

In this review, we discuss the recent evidence on clinical, molecular and epidemiological features of VTE in older frail subjects focusing on the patients with PD and parkinsonism. We also discuss some therapeutic issues about the risk prevention and we suggest comprehensive geriatric assessment (CGA) as the optimal strategy to identify and prevent the VTE risk in these patients.

## 2. VTE in Older Patients: Results from Epidemiologic Studies

VTE has a significant clinical and epidemiological impact on the elderly. The VTE incidence increases to more than 1% per year in older patients, suggesting the presence of specific age-related risk factors in this population [2].

The Worcester VTE Study, which analyzed the medical records of all residents from Worcester (MA, USA) who had a VTE diagnosis according to the International Classification of Diseases, 9th revision (ICD-9), found a total of 587 patients with VTE, with an incidence and attack rates of 104 and 128 per 100,000 subjects, respectively. The authors found also that the prevalence in older patients was more than 60% among all VTE cases, and that there was a 10-fold increased incidence in subjects aged >75 years, when compared with those aged <55 years [3,4]. 

Following these results, a prospective study that prospectively followed 542 patients aged ≥65 years with VTE from January 2008 through August 2011, found that advanced age is associated more often with provoked, malignancy and hospital-associated VTE unlike the VTE observed in the general, younger population. Interestingly, the authors found that the 22% of VTE patients with a recent hospitalization or surgical intervention received no antithrombotic prophylaxis, and that around 1/3 of patients aged between 65 and 74 years suffered from a malignant condition. In the older patient category, age extremities (≥80 years) seem not to influence the VTE recurrence but are associated with a higher risk of all-cause mortality, and with a greater bleeding risk due to an excessive anticoagulant therapy. This seems to be reasonable considering the concomitant presence of renal and liver dysfunction of many older patients receiving anticoagulants. Although the co-existence of comorbidities might influence the risk of mortality, VTE and its treatment may significantly influence this risk by leading to the death of more than one quarter of very old (≥80 years) patients, as demonstrated in a large perspective study conducted on more than 20,000 patients [5,6].

## 3. Etiology of VTE in Older Patients

Several inherited and acquired disorders can be associated with VTE. In the elderly, patients with VTE are more frequently women, have suffered a recent immobilization from fracture or heart failure, and have had more often a provoked (in-hospital) rather than unprovoked VTE event [6]. 

Table 1 shows the most frequent causes and molecular mechanisms of VTE in the elderly. Several causes can be associated with an increased VTE risk such as immobilization syndromes, malignancies, congestive heart failure, diabetes mellitus, chronic obstructive pulmonary disease, stroke and genetic factors, with different mechanisms for each cause [2,7,8,9,10].

However, there is no singular cause or mechanism that increases the risk of VTE in the elderly and more clinical and molecular factors can play an important thrombogenic action. Older patients have indeed several conditions which can promote the VTE itself (e.g., previous fracture, heart failure, immobilization and conditions characterized by chronic inflammation that can lead to a persistent endothelial dysfunction, hypercoagulabity and thrombotic events) and therefore it is difficult or downright impossible to distinguish the leading etiology in most cases, especially in those cases where there is an inherited predisposition to VTE [11]. Chronic inflammation in older patients is considered the main mechanism that increases the risk of thrombotic events in the elderly, and therefore its molecular processes are here discussed. Regardless of the etiology, the leading theory about the pathogenesis remains the complex interplay between the three components of the Virchow’s triad, also at a molecular level, acting differently in the elderly due to the presence of different clinical backgrounds and risk factors.

## 4. Aging-Related Inflammation and Consequent Risk of Thrombosis: Molecular Mechanisms 

### 4.1. Chronic Inflammation in the Elderly (Inflammaging)

Although acute inflammation is a well-known phenomenon that can be beneficial against pathogenic noxa, low-grade but persistent inflammation can be associated with the onset of specific diseases. In the elderly, the presence of persistent inflammation without an overt infection (so-called inflammaging), is significantly associated with an increased risk of morbidity and mortality, in particular, related to thrombotic events [12,13].

The specific molecular mechanisms are complicated and largely undiscovered. Chronic inflammation shares several mechanisms with an acute response to an injury but it is not time limited despite its lower grade, resulting in tissue degeneration and development of many diseases.

Several authors hypothesized that specific sources can trigger inflammaging, such as the accumulation of damaged cells and cellular waste products (e.g., extracellular ATP free radicals, peroxidized lipids, cardiolipin, urate products and fatty acids) that can mimic exogenous molecules that stimulate the innate host response [12,14].

Another active source of inflammaging could be represented by the age-related release of mitochondrial “damage”-associated molecular patterns (DAMPs) from the activation of the nucleotide-binding and oligomerization domain-like receptors (Nlrp3) inflammasome that is a macromolecule capable of stimulating pro-caspase-1 after exposition to pathogenic noxa. This process results in an increase production of mitochondrial reactive oxygen species that promote innate immunity being exceptionally similar to bacterial molecular products [15].

Furthermore, an aging-related change of gut microbiota and a reduced capability of the gut in the elderly to sequester bacterial products could be associated with increased gut mucosa permeability and persistent toxic leakage into the circulation [13,16]. For example, the aging gut microbiota may be associated with increased systemic absorption of bacterial lipopolysaccharide (LPS), prompting subclinical immune activation and chronic inflammation [13,16] 

Age-associated immunologic rearrangements, both expressed as a reduced adaptive and mild increased innate activity and also as a compromised complement function, can contribute further to the inflammaging process, regardless of the presence of pathogenic (autoimmune and exogenous) stimuli [17]. 

**Figure 1:**
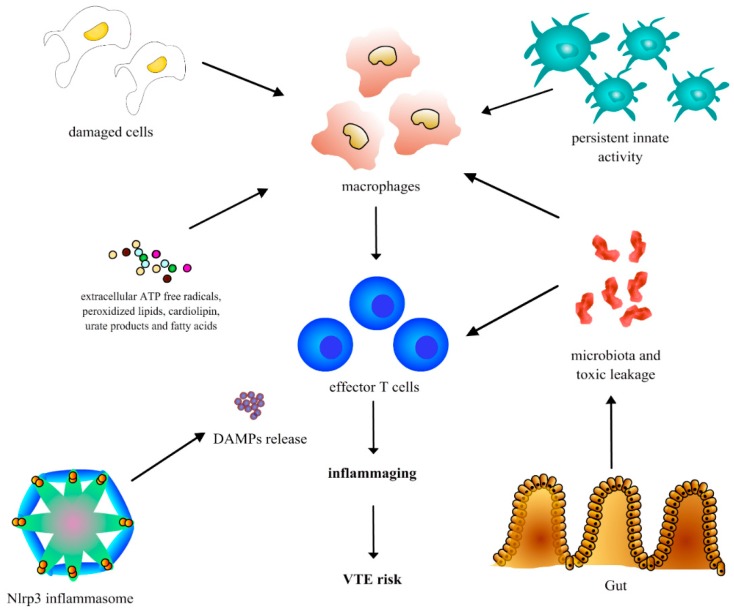
The main molecular mechanisms that could lead to chronic inflammation in the elderly and risk of VTE.

As a further step, persistent inflammation could result in an increased hypercoagulation status and risk of arterial and venous thrombosis in the elderly [12]. In particular, the persistent synthesis of proinflammatory chemokines and cytokines could promote the tissue factor (TF) production from monocytes and activate the coagulation cascade. Furthermore, chronic inflammation can reduce the production of molecules such as protein C and antithrombin III (AT-III) and compromise their anticoagulant activity. Other mechanisms include vessel wall damage induced by chronic inflammation, that is associated with impairment of antiaggregant, anticoagulant and vasodilatory characteristics. In particular, endothelial damage is associated with the release of cytokines (e.g., IL-1α and β), chemokines and growth factors, and consequent platelet activation, aggregation, and with a reduced thrombomodulin secrection, that is associated with a lower capability of activating the anticoagulant protein C and inhibiting thrombin [18]. Furthermore, neutrophils seem to be emerging players in the induction and propagation of thrombosis, in particular the interaction between endothelial cells with leukocytes and platelets is associated with an increased TF production and activation of coagulation cascade, and the release of proinflammatory mediators from neutrophils and endothelium is associated with muscular smooth cells proliferation, that along with the production of platelet activating factor (PAF) and endothelin-1 is associated with a significant impairment of vasodilator activity. Finally, hypercoagulation can induce further inflammatory activity, which is associated with additional thrombotic risk in a vicious process [18]. The main molecular mechanisms that could lead to chronic inflammation in the elderly and the consequent risk of VTE are shown in Figure 1.

### 4.2. Chronic Inflammation, Oxidative Stress and Parkinson’s Disease

Despite that it is well recognized that intraneuronal accumulation of protein α-synuclein and loss of dopamine neurons in the substantia nigra are the main pathologic features of PD, the causes of this process are still unknown. Regardless of the etiology, there is increasing evidence that chronic inflammation in the nervous system (neuroinflammation) and oxidative stress play a key role in the pathogenesis of the disease [19]. Although evidence is limited, it can be postulated that the persistence of neuroinflammation in older patients with PD could contribute not only to the neuronal degeneration and to the classical clinical manifestations of disease but also to the thrombotic risk [20]. 

The α-synuclein accumulation, together with aging and genetic factors, promotes and maintains the inflammatory response and consequent oxidative stress that is responsible for neuronal degeneration and neuron loss. In particular, these factors activate microglia and astrocytes to release cytokines and reactive oxygen species (ROS) that recruit peripheral leukocytes into the central nervous system (NS). If on one side inflammation serves to clear damaged and cells and abnormal debris, on the other, it contributes to neuron death and loss, that stimulates inflammation and further cell death in a vicious process [21].

The recent knowledge about the pleiotropic functions of microRNAs, a group of small endogenous non-coding molecules able to alter the mRNA transcription and post-transcriptional gene processes in several chronic diseases involving not only the central nervous system [22], and the evidence that microRNAs can reduce oxidative stress by modulating reactive oxygen species (ROS) accumulation in dopamine neurons, raises the hypothesis of specified mechanisms that could be a target for tailored therapies in the future, with the aim to reduce not only the neuronal damage but also chronic inflammation, oxidative stress and related thrombotic risk [19]. 

## 5. The Complex Molecular Interplay between the Three Components of the Virchow’s Triad and the Influence of Aging

### 5.1. Vascular Endothelial Dysfunction 

Endothelial dysfunction (ED) is considered the first alteration that occurs after a vascular injury and activates a cascade of molecular processes that lead to the formation of atherosclerotic plaque such as vasospasm due to a low concentration of nitric oxide (NO), increased platelet adhesivity, vascular hyperproliferation and coagulation disturbances [23]. 

Although the atherosclerotic plaque formation has been considered for several years a disorder far from the venous thromboembolism, there is growing evidence that these disorders can co-exist and that VTE can be associated with early atherosclerotic changes and then with symptomatic plaques [23]. 

Prandoni et al. have indeed demonstrated a 60% higher risk of developing symptomatic atherosclerotic plaques in older patients with VTE of unknown origin as compared to patients with secondary VTE [24]. In another study, Jezovnik et al. found a higher prevalence of atherosclerotic plaques in patients with VTE than in age-matched controls (33/47 vs. 15/44, *p* < 0.001) but also a higher intima-media thickness (IMT) in patients than in controls (0.94 mm ± 0.29 vs. 0.71 mm ± 0.15, *p* < 0.001), confirming the hypothesis of coexistence of preclinical atherosclerotic changes in patients with VTE [25].

The classic evidence that endothelial dysfunction and atherosclerotic plaque increase with age and that both conditions (atherosclerosis and VTE) can be found more frequently in the elderly reinforce the hypothesis that the two diseases can share similar mechanisms [26].

In addition, PD patients show chronic high levels of pro-inflammatory cytokines that could be associated with persistent ED and therefore with a significant thrombotic risk. King et al. found that PD patients demonstrate a significant increase in blood levels of inflammatory cytokines such as TNA-α, IL-6, IL-10 and IL-1β. The authors also found a significant C-reactive protein (CRP) elevation in PD patients, confirming the presence of a chronic inflammatory background that could predispose them to VTE [27].

The ultrasound evaluation of the flow-mediated dilation (FMD) of the brachial artery is useful to assess the presence of ED in patients with risk factors for atherosclerosis such as metabolic syndrome, hypertension and diabetes, by revealing the presence of a significant FMD impairment [28]. 

In elderly patients, the FMD method was equally effective as compared to invasive methods and should be preferred for its higher feasibility and ease of use [29]. Several authors have demonstrated that there is a significant FMD reduction in patients with VTE. In particular, Mazzoccoli et al., measured the FMD in 60 older patients with and without idiopathic DVT, matched for cardiovascular risk factors. Interestingly, DVT remained an independent predictive factor of low FMD when compared to other parameters in a multivariate analysis [30]. Conversely, epicardial fat (EF) thickness and FMD values were respectively lower and higher in patients without DVT, when compared to those with DVT (9.07 ± 1.89 mm vs. 12.32 ± 1.73 mm, *p* = 0.005, and 9.01 ± 2.77 percent vs. 7.47 ± 5.37 percent, *p* = 0.058 respectively), confirming the presence of pre-clinical markers of atherosclerosis in patients with VTE [31].

Recent in vitro studies support the evidence of the presence of ED in VTE patients; some authors investigated the endothelial colony-forming cells (ECFCs) properties of patients with VTE and found a significant dysfunction of them with an early appearance (7 vs. 21 days), a higher CD34 expression and significant abnormalities in the mitochondrial membrane when compared to the controls. [32].

Furthermore, the authors found that ECFCs from VTE patients had an impaired *ephrin-B2/Eph-4* gene transcription and enhanced oxidative stress with increased ROS, suggesting also a higher prothrombotic oxidation-related risk in these patient as compared to controls [33].

### 5.2. Hypercoagulable Conditions

Genetic causes of thrombosis are not common. Although most evidence demonstrates that hypercoagulation derived from genetic risk factors plays an important role in increasing the VTE risk in younger patients, there is increasing evidence of an influence in the elderly, in particular from factor V Leiden and prothrombin-PT-G20210A mutation, non-O blood group and family VTE history. 

Factor V Leiden is a mutated form of the human factor V that is resistant to the normal inhibition from the anticoagulant proteins. Homozygous V Leiden is extremely rare in the general population, and most frequently the mutation manifests as heterogenous. The presence of factor V Leiden leads to a state of chronic hypercoagulability and, therefore, to an increased thrombotic risk. Also, other heterogenous genetic defects can be found as co-founders of thrombosis, rather as the main causative agent, such as the prothrombin (PT) G20210A mutation, where there is guanine to adenine substitution at the 20210 DNA gene position that leads to an enhanced activity of the coagulation cascade. The case-control study of Karasu et al., which investigated the association between the most common genetic risk factors and the VTE risk in 401 patients vs. 431 controls (all ≥70 years old), found that a positive family history had a positive impact on the risk of VTE, and that the hazard was increased 2.2 times if the patients were carriers of the Factor V Leiden variant (95% CI, 1.2–3.9), 1.4-fold if they had the PT mutation (95% CI, 0.5–3.9) and 1.3 if they had the non-O blood group (95% CI, 1.0–1.8) [34].

Although hypercoagulation derived from an unbalanced level of hemostatic factors such as fibrinogen, D-Dimer, Factor VIII and Von Willebrand Factor (VWF) is associated with an increased risk of VTE in the general population, there is lower evidence in older patients, in which a significant VTE risk has been documented only in patients with high Factor VII, VIII and VWF levels, in particular for factor VIII, the hazard ratio (HR) was 2.6 (95% CI: 1.6 to 4.3) in the highest quartile and 3.8 (95% CI: 2.0 to 7.2) for the highest fifth percentile; for von Willebrand factor, HR was 4.6 (95% CI: 2.2 to 9.2) for the highest quartile and 7.6 (95% CI: 3.1 to 18) for the highest fifth percentile; for Factor VII levels above the 95th percentile, HR was 2.4 (95% CI: 1.2 to 4.8) [35].

In murine models of aging, acute inflammation is associated with hypercoagulability, also through a significant elevation of plasminogen activator inhibitor-1 (PAI-1), an important inhibitor of fibrinolysis that is mainly secreted the endothelium. An excessive PAI-1 elevation could result in an inappropriate blocking of the active site of urokinase plasminogen activator (uPA), and therefore an under-stimulation of the lytic process of plasminogen to plasmin. A reduced plasmin activity is associated with lower fibrin degradation and clot accumulation [36]. 

More certainly, a significant prothrombotic risk in the elderly can occur with platelet dysfunction, which increases during aging and can contribute to the VTE risk. In the elderly, platelet activation, aggregation, and secretion are overactivated as compared to the younger population. In particular, older subjects show higher plasma levels of platelet factor 4 (PF4) and beta-thromboglobulin (β-TG), that are fundamental mediators in platelet activation, lower thresholds of platelet aggregation to the adenosine diphosphate (ADP) trigger and reduced bleeding time, an indirect marker of enhanced clot aggregation, Furthermore, older subjects also show higher prostacyclin metabolite secretion and resistance to the inhibition from prostaglandin I2 (PGI2), a molecule that acts by promoting vasodilation and blocking aggregation of platelets [37].

The hypercoagulation-related prothrombotic risk could derive also from therapies that are prescribed in this population, in particular in PD patients. It has been demonstrated that the treatment with levodopa (l-DOPA) could increase the serum levels of homocysteinemia. l-DOPA is metabolized mainly to 3-*O*-methyldopa in a methylation reaction in which *S*-adenosylmethionine donates a methyl-group and is converted to *S*-adenosylhomocysteine. *S*-adenosylhomocysteine is then hydrolyzed to form homocysteine [38]. Hyperhomocysteinemia is associated with overinhibition of thrombomodulin, which cannot activate the anticoagulant protein C, and is therefore associated with a 2–3-fold higher VTE risk when compared to the hazard observed with normal values. In the PD population, a significant elevation of homocysteinemia therapy-related could significantly influence the risk of VTE [39]. Despite that APS is an important acquired cause of thrombosis in young patients, it has been postulated that it could have an influence on the elderly. In particular, it has been demonstrated that the prevalence of anticardiolipin antibodies (aCL) increases with age. Furthermore, several conditions that are typical of the elderly, such as MGUS (monoclonal gammopathy of uncertain origin), the presence of rheumatoid factor, multiple and long-term drug administration, kidney and liver disease, polymyalgia rheumatica, solid cancers and lymphomas can be associated with significantly higher levels of antiphospholipid antibodies (aPL) [40]. Despite these theories, there is an overall low evidence of the specific role of genetic causes of thrombosis in the elderly, and no screening recommendations can be made on the basis of these limited data.

### 5.3. Functional Limitation, Muscle Strength Reduction and Blood Stasis

It is well documented that immobilization is associated with a high thrombogenic burden by increasing blood viscosity and stasis secondary to a prolonged bed rest [2,41]. Functional limitation and reduction of muscle strength, by affecting the calf muscle pump action, could lead to a reduced blood venous compliance with increased reflux and stasis, and consequently to a higher risk of thrombosis [2]. Furthermore, calf muscle pump compliance, capacitance and consequent pressure transmission decline significantly with aging, and these alterations could further promote the risk of thrombosis in elderly patients [42].

Beyond the calf muscle pump dysregulation that acts as a trigger for thrombotic events, chronic inflammation could itself mediate the risk of VTE in older patients through the functional limitation. Several studies have found that elderly patients, particularly women, with high levels of cytokines and inflammation markers such as IL-6 and CRP, had lower muscle strength and higher walking impairment than those with reduced serum levels [43,44,45,46,47].

The catabolic effect of inflammatory cytokines such as tumor necrosis factor (TNF)-α on muscle, expressed as a significant whole-body protein breakdown, muscle atrophy and protein degradation, is related to a significant cytokine production and contributes to the functional limitation and disability that occur over time in elderly patients [47]. In in vitro studies, TNF-α and IL-6 infusion in rats induces myofibril loss, protein degradation and consequent muscle atrophy [48,49].

The catabolic effect of chronic inflammation on muscle decline of function goes beyond the direct action on cellular senescence and oxidative stress. An increased cytokine receptor activity, as demonstrated by the presence of higher levels of soluble tumor necrosis factor receptor (sTNFR) in the serum of elderly patients, could contribute further to the inflammaging-related functional disability by enhancing cytokine signaling and protein breakdown [50]. 

The evidence of the functional impairment and consequent risk of thrombosis in older patients is supported by several clinical studies. A recent meta-analysis has confirmed that immobilization is associated with a significant risk of VTE events in all subgroups of studies (overall Odds Ratio -OR-: 2.52; 1.70–3.74; *p* < 0.001). However, there was no specific information on age classes or how the immobilization could influence *per se* the VTE risk [51]. 

The results from a prospective population-based study have confirmed that conditions that increase the VTE risk in the general population, such as surgery or recent fracture, are associated with a higher risk of VTE events in subjects ≥65 years old (*p* = 0.0002 and 0.03 respectively). However, even if it is reasonable to hypothesize that these risk factors could increase the thrombotic risk by leading to a significant immobilization, no specific information on the definition and characteristics of the immobilization period itself were present in the study [6]. 

A case-control, multicenter and hospital-based study with prospective data collected on geriatric university hospitals with long-, intermediate-, and short-term care facilities, found that the mobility restriction, defined as limited mobility without immobilization or immobilization in bed <15 days, was independently associated with the development of VTE in older patients (OR 2.1, 95% CI 1.39–3.17, *p* < 0.0006 and 6.67, 95% CI 2.97–14.99, *p* < 0.0001, respectively) [52]. Engbers et al. found that transient immobility during hospitalization and out of hospital was associated with a significantly high risk of VTE in adults aged > 70 years old over the next 3 months after discharge (OR 5.0, 95% CI 2.3–11.2), suggesting that the preventive measures of VTE events should not focus only on the hospital but also the home setting [53].

Despite robust evidence that immobilization significantly influences the risk of VTE in older patients, there is a lack of evidence about the specific influence of the singular disorders that cause immobility in this population, in particular in patients affected by advanced stages of PD and AD. In an observational study we investigated the mobility, walking ability (by using a 5-item standardized scale) and (medical and surgical) risk factors for VTE in 140 patients with PD (aged 65 or older) admitted in our Geriatric Department over the period November–December 2008. We found that nine (6.4%) patients were affected by thromboembolism (eight with acute EP and one with DVT of the legs). According to the Hoehn and Yahr Staging scale, which evaluates the disability associated with advanced stages of PD, we found that three of them were bedridden, three confined into a chair and three were able to walk with assistance. None of them were taking preventive drugs such as heparin [54].

More recently, Engbers et al. found a 2.3 times higher VTE risk (OR = 2.3, 95% CI = 1.5–3.4) in patients with low handgrip strength, 2.9 in patients with impaired activities of daily living (ADLs, OR = 2.9, 95% CI = 1.6–5.3), 3 in those with reduced mobility (OR = 3.0, 95% CI = 1.9–4.7) and 4 in sedentary individuals (OR = 4.0, 95% CI = 2.5–6.3) [55].

Furthermore, the co-existence of other factors such as varicose veins, leg ulcers and oedema can further significantly increase the risk of VTE (OR: 10.5; 95% CI 1.3–86.1) [56].

## 6. The Comprehensive Geriatric Assessment as a Useful Tool for VTE Risk Identification 

In this setting, a thorough clinical evaluation, aimed at assessing the motor capability of the patient, physical function, balance and muscle strength, could be useful to predict the VTE risk. 

In detail, we proposed that a brief evaluation of the cognitive, motoric and subtle neurological signs could be used in clinical practice in advanced stages of PD and older persons with parkinsonism to identify subjects with reduced mobility, cognitive impairment and subsequent high risk of VTE. Older subjects, with or without parkinsonism, who scored less than 10 in the Short Physical Performance Battery (SPPB) [57] and with executive dysfunction evaluated for example by the Clock drawing test (CDT) [58] and with subtle neurological signs [59], such as the presence of the Babinski sign, cogwheel rigidity or palmomental reflex could identify older persons with “multiple neurological dysfunction” with reduced physical performance and at risk of disability and at high risk of VTE.

The SPPB score is extensively validated worldwide and in different medical settings to identify frail older persons, and it is capable of predicting negative outcomes such as hospitalization, falls and admission to nursing home and dying [60], despite multimorbidity of the subjects [61]. In patients with parkinsonism, an objective measure of the physical performance could be particularly useful to predict falls and immobilization with high risk for VTE [62].

In many studies, the clock drawing test (CDT), which is a cognitive test that could detect executive dysfunction despite a normal score of the mini mental score examination (MMSE) [58], explores globally cognitive function but not specifically executive performance of the subjects. Given that in PD and parkinsonism, the early modifications of the cognitive status are executive dysfunctions, it appears reasonable that the CDT could be included in the initial global evaluation of older persons as part of its comprehensive geriatric assessment (CGA).

Finally, subtle neurological signs are particularly prevalent [63] in older persons and their presence could be interpreted as the presence of multiple neurological disease related to aging, such as PD plus chronic vascular lesions related to hypertension [64], or normal pressure hydrocephalus associated with vascular lesions. Therefore, these chronic neurological diseases in older persons could change the trajectory of an isolated neurodegenerative disease if they are present simultaneously in older subjects [45]. Interestingly, postural abnormalities are associated with an increased DVT risk in PD patients. Yamane et al. demonstrated a significant association between the bent knee posture and risk of leg thrombosis [65]. These data confirm how a comprehensive medical examination is important to evaluate PD patients not only for assessing neurological symptoms but also to predict the VTE risk.

We proposed this “3×3-CGA” evaluation, which is composed of the SPPB score to describe the physical performance of older persons with parkinsonism, the 4-AT, GDS and CDT for identifying persons with executive dysfunction and a quick neurological examination to capture subtle neurological signs, such as the Babinski sign, cogwheel rigidity and palmomental reflex, that globally describe patients with neurologic disorders such as parkinsonism, and with reduced motoric, cognitive performance and therefore at risk of negative outcomes such as VTE. 

The “3×3-CGA” assessment could add clinical information for older persons with neurological diseases and associated reduced cognitive and physical performance. This information could be useful in different (not only geriatric) settings, such as after discharge from the Emergency Department, for outpatient evaluation, and in all Geriatric and Internal Medicine Units, given that older persons are “the core business” in our Hospital due to the growing epidemiological trend of the aging process.

The “3×3-CGA” could therefore identify older persons with unknown parkinsonism and at high risk of negative outcomes, such as falls, immobilization, aspiration pneumonia and subsequent disability and possible VTE with fatal events. Interestingly, in the current state of art, the utilization of antithrombotic drugs (such as antiplatelet therapies) is not standardized in older persons when subtle neurological signs are present (consistent expression of diseases) and even in the routine prescription of anticoagulants in the prevention and treatment of VTE (both DVT and PE).

**Figure 2:**
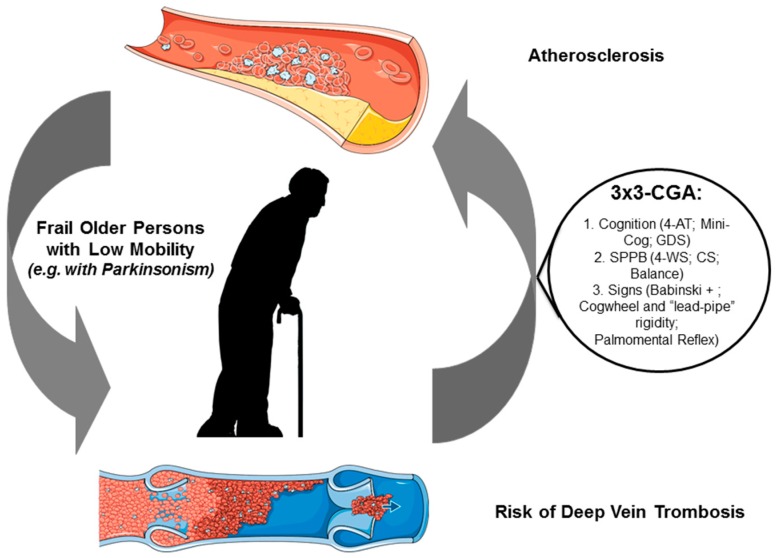
Main mechanisms that could increase the VTE risk in older individuals. The mini-CGA could be useful to identify older persons with unknown parkinsonism and at high risk of negative outcomes, such as falls, immobilization, aspiration pneumonia and subsequent disability and possible VTE with fatal events.

Figure 2 summarizes the main mechanisms that could increase the VTE risk in older individuals and shows our proposal for the VTE prevention according to the ”3×3-CGA”.

## 7. Risk Prevention in the Elderly: Proposals for Anticoagulant Strategies

### 7.1. Risk-Benefit Ratio of Thromboprophylaxis in Older Patients

Antithrombotic treatment in older patients represents a great therapeutic challenge. Given the frail condition of these patients, and often the co-existence of liver and kidney disease that could overexpose the patients to increased drug concentrations and the higher bleeding risk in comparison to that of younger individuals, antithrombotic treatment should be prescribed with caution after a careful evaluation of the risk-benefits ratio in each patient, in terms of overall survival, improvement of quality of life and short- and long-term risk of complications [66,67]. More often, the routine evaluation of older patients that are admitted to medicine and geriatric wards after a fall, reveals the presence of bleeding that could be often avoidable with a rational modulation of an inappropriate out and in-hospital antithrombotic prescription [68]. On the other hand, given the higher VTE risk of older individuals with at-risk conditions such as CHF, COPD, DM, stroke and age-specific risk factors such as immobility and age-related hypercoagulation, an appropriate VTE prevention strategy should be delineated to avoid the risk of thrombotic events. Furthermore, a tailored approach should be used in older patients to treat conditions such as DVT and PE with the aim of achieving the lowest bleeding risk with a sufficient therapeutic effect [68].

### 7.2. VTE Prevention in Older Patients

The American College of Chest Physicians (ACCP) guidelines recommend preventive anticoagulation in hospitalized non-surgical patients at high risk of thrombotic events, in particular those with a Padua Prediction Score ≥4, where the risk points include the presence of active cancer (3 points), previous VTE (3), reduced mobility (3), already known thrombophilic condition (3), recent (≤1 month) trauma and/or surgery, elderly (≥70 years old, 1) and other conditions such as heart and/or respiratory failure, stroke, myocardial infarction, obesity with BMI ≥ 30, sepsis, acute rheumatologic disorder and treatment with hormones (one point each). In these patients, the panel expert recommends thromboprophylaxis with drugs such as low-molecular-weight heparin (LMWH), low-dose unfractionated heparin (LDUH) twice or three daily or fondaparinux (Grade 1B) [69,70]. 

Immobilization and age, in particular in patients with bed rest longer than a period of three days, are critical risk points because they represent an indication of thrombopropylaxis, especially if one (immobilization) or three additional other points (age) are simultaneously present in the same patient. In this setting, the immobilization associated with conditions such as Parkinson’s disease and parkinsonism in older hospitalized patients raises a significant higher risk for VTE that should be promptly identified and prevented consequentially, also if patients do not have an acute medical illness [70].

The dosage of the therapy, according to the different drugs should be modified appropriately in the presence of severe kidney disease (glomerular filtration rate (GFR) less than 30 mL/min/m^2^) to avoid the risk of drug accumulation, significant bleeding and mortality [71].

In older patients, the correct choice of thromboprophylaxis should be based on the patient compliance and ease of administration, taking into account the patient, caregiver and/or family preference because this can influence the success of the therapy [72].

The ACCP guidelines recommend that patients that are at significant bleeding risk for major events should undergo mechanical thromboprophylaxis if they are at higher risk of thrombosis too, despite less consistent evidence (Grade 2C) for both graduated compression stockings or intermittent pneumatic compression [70]. 

Balance instability, cognitive impairment and frailty conditions should be evaluated carefully before prescribing thromboprophylaxis because this could increase the risk of falls and therefore the risk of significant bleeding, in particular in those with multiple comorbidities [71,72,73,74,75,76,77,78,79,80].

### 7.3. Tailored Approaches for the Treatment of VTE in the Elderly

Anticoagulants should be started promptly after evidence of VTE (DVT or PE), unless contraindicated. Older patients, in particular those with PD or parkinsonism, could have a different dose requirement (both LMWH and warfarin), according to dietary habits, kidney and liver function. This justifies specified approaches of anticoagulation in the elderly, taking into account the nutritional status, frailty condition and presence of organ damage to reduce the risk of bleeding [81,82,83,84].

In particular, patients with severe kidney impairment (GFR less than 30 mL/min/m^2^) should receive a half dose according to the body weight in order to reduce the risk of bleeding due to an increase in drug concentration [85]. 

The presence of neurogenic dysphagia in patients with PD or parkinsonism often alters daily feeding, especially in association with a low caloric, protein and vitamin intake and with a decreased nutritional status. A low vitamin K intake overexposes patients to excessive drug concentrations, which require more frequent dosage evaluation and visits to reach stability over time. In order to reduce the international normalized ratio (INR) lability after warfarin administration, some authors have suggested an oral vitamin K supplementation in patients with unstable anticoagulation due to reduced vitamin intake. In their study, the supplementation was associated with a higher INR stability compared to INR of controls treated with placebo (28% ± 20% vs. 15% ± 20%; *p* < 0.01) [86].

### 7.4. Advantages and Limitations of Novel Oral Anticoagulants in the Elderly

The relatively recent introduction of novel oral anticoagulants (NOACs) is changing the therapeutic scenario in these patients because they have demonstrated a safer profile than conventional anticoagulation therapy, with a concomitant reduction of recurrent VTE in at-risk patients [87,88].

In particular, a pooled analysis of the EINSTEIN-DVT and EINSTEIN-PE trials, which compared the safety/efficacy profile of rivaroxaban versus conventional antithrombotic therapy (enoxaparin followed warfarin or acenocoumarol) demonstrated a significantly lower incidence of major bleeding in fragile patients when treated with rivaroxaban as compared to those treated conventionally (HR: 0.27; 95% CI: 0.13 to 0.54) [89]. Similar results were obtained with edoxaban versus warfarin in fragile patients [90], confirming the favorable risk/benefit ratio of NOACs in this population versus standard therapy. 

According to these results, NOACs such as rivaroxaban can be used in fragile patients with a significant risk of bleeding who necessitate an appropriate anticoagulation to reduce the risk of recurrent VTE [87].

However, there are some disadvantages and limitations of using NOACs in the elderly. In particular, older patients present often moderate to severe renal failure and can be exposed to excessive blood drug concentrations related to a lower drug elimination. Therefore, NOACs should be used with caution in this setting, and a dose adjustment is required for rivaroxaban and apixaban, while dabigatran is contraindicated in patients with severe kidney disease because is mainly metabolized from the kidney [87]. NOACs are characterized generally by a shorter half-life as compared to warfarin. While this feature can be useful to limit the severity and time of bleedings, a reduced compliance (for example, related to the presence of cognitive impairment and a lack of adequate caregiver supervision) and secondary drug interruption can be associated with an increased thrombotic risk [87]. Finally, another limit of NOACs in the elderly is that there is low evidence of effectiveness in patients with cancer; therefore, no definitive conclusions can be made in this category [90].

## 8. VTE Prevention and the Pleiotropic Effects of Acetylsalicylic Acid and Other Antiplatelet Drugs

The role of platelets in determining VTE is unclear, despite the well known contribution they have in the pathophysiology of arterial thrombosis. Few studies (nine) have investigated the effectiveness of acetylsalicylic acid (ASA) and other antiplatelet drugs for VTE prevention, despite their clinical advances in special populations (e.g., elderly), such as no need for effective monitoring, ease of administration (oral route), and low cost [70]. An overview of the results showed that antiplatelet agents were associated only with a reduced hazard of asymptomatic DVT (RR, 0.65; 95% CI, 0.45–0.94), but not with significant effects on PE incidence [91]. 

The Embolism Prevention (PEP) Trial investigated the effect of ASA/antiplatelet drugs on VTE incidence and safety profile in more than 13,000 patients undergoing hip surgery for fracture. Compared to the placebo, the authors found a reduced incidence of PE with ASA (43%, 95% CI 18–60; *p* = 0.002) and DVT (29%, 95% CI 3–48; *p* = 0.03). Similar effects were obtained with the other treatment approaches. However, the authors did not find any significant difference in terms of mortality from other vascular (HR 1.04 (95% CI 0.86–1.26)) or non-vascular causes (1.01 (0.84–1.23)) and there were no significant differences in bleeding rates [92].

Following these results, the ACCP guidelines do not recommend ASA/antiplatelet drugs in VTE prevention in hospitalized patients, suggesting further studies aimed at investigating the VTE risk reduction with antiplatelet drugs [70].

However, the use of ASA/antiplatelet drugs could be of benefit in older patients with PD or parkinsonism beyond the direct effect on the VTE risk. These patients often have brain lesions due to ischemic (acute or chronic) vascular injures [93], and the prevalence of vascular lesions is high in this population, suggesting that in the vascular parkinsonism subtype, which is attributable mainly to subtle and multiple clinical reasons, recurrent ischemic lesions can be associated with the onset of ischemic injuries [94].

Some authors have indeed found a higher risk of ischemic stroke in patients affected by PD (HR of 2.37, 95% CI 1.92 to 2.93, *p* < 0.0001) when compared to controls. Furthermore, they found a lower survival rate free from stroke in PD vs. non-PD patients (*p* < 0.0001) [95].

There is evidence of a protective effect of ASA/antiplatelet drugs in these patients. It has been hypothesized that nonsteroidal anti-inflammatory (NSAIDs) drugs may reduce the risk of PD onset by blocking several neuroinflammatory pathways [96,97]. 

If these results can be confirmed, the positive effects can be significant. Although evidence of a VTE risk reduction with ASA/antiplatelet drugs is limited, we hypothesized that this risk improvement can derive mainly from a reduced incidence of ischemic stroke and related negative outcomes such as cognitive impairment, paralysis and reduced motoric function and immobilization [98,99,100,101].

## 9. Conclusions

The risk of thrombotic events in elderly patients represents a relevant issue. This is true for those with PD or parkinsonism, even if a surprisingly low number of studies have specifically assessed the association between PD and thrombosis. There is evidence that VTE has a significant impact in terms of mortality and function loss in older patients, and this may also apply to those with PD and parkinsonism. A multidisciplinary and comprehensive approach is therefore suggested to assess the risk of thrombotic events, by evaluating not only the presence of clinical (medical) risk factors but also the cognitive, motoric and subtle neurological signs that could influence the risk. Anticoagulants should be chosen appropriately on the basis of a global evaluation of the elderly patient that includes a tailored cost-benefit ratio assessment, in order to avoid any risk of bleeding.

Considering the evidence of some benefits of ASA/antiplatelet drugs in VTE risk reduction due to multiple mechanisms that go beyond the simple antithrombotic function, there is a need for large prospective studies aimed at assessing the positive effects of these drugs in the elderly, in particular in the PD and parkinsonism population.

## Figures and Tables

**Table 1:** Most frequent causes of venous thromboembolism (VTE) in the elderly.

Etiology	Molecular Mechanisms of Thrombotic Action	Prevalence (%)	Ref
Malignancy	Hypercoagulation, blood stasis	10	[6]
Immobilization	Blood stasis	25	[7]
CHF	Hypercoagulation, endothelial dysfunction, blood stasis (advanced stages)	22	[7]
DM	Hypercoagulation, endothelial dysfunction, blood stasis	16	[8]
COPD	Hypercoagulation, endothelial dysfunction	11	[9]
Genetic risk factors	Hypercoagulation	7	[2]

CHF: Congestive heart failure; DM: Diabetes mellitus; COPD: Chronic obstructive pulmonary disease.
